# Rapid screening of acute promyelocytic leukaemia in daily batch specimens: A novel artificial intelligence‐enabled approach to bone marrow morphology

**DOI:** 10.1002/ctm2.1783

**Published:** 2024-07-23

**Authors:** Yujun Xiao, Zheng Huang, Jun Wu, Yan Zhang, Yuanyuan Yang, Chun Xu, Fangyu Guo, Xiong Ni, Xinhua Hu, Jianmin Yang, Yang Song, Hui Cheng, Gusheng Tang

**Affiliations:** ^1^ Department of Hematology Changhai Hospital Naval Military Medical University Shanghai China; ^2^ School of Health Science and Engineering University of Shanghai for Science and Technology Shanghai China; ^3^ Department of Materials Science and Key Laboratory of Micro‐ and Nano‐Photonic Structures (Ministry of Education) Fudan University Shanghai China; ^4^ Nursing Department Changhai Hospital Naval Military Medical University Shanghai China; ^5^ Cellsee (Wuxi) Intelligent Technology Co., Ltd Wuxi China; ^6^ Department of Hematology Changzheng Hospital Naval Military Medical University Shanghai China

Dear Editor,

Acute promyelocytic leukaemia (APL) is a malignant haematological disease characterised by abnormal proliferation of promyelocytes and represents a distinct subtype of acute myeloid leukaemia (AML), constituting about 15% of AML cases.^1^ According to real‐world data, the early mortality rate for APL varies between 17% and 40%.^2^, ^3^, ^4^ In order to avoid premature deaths, rapid and accurate diagnosis is crucial for early identification and initiation of treatment with all‐trans retinoic acid (ATRA) and arsenic trioxide (ATO) or chemotherapy.^5^ Although definitive diagnosis of APL requires confirmation of chromosome t (15;17) or *PML::RARA* fusion gene,^6^ cytomorphology remains the fastest technique for initial diagnosis.

Manual microscopic examination of cytomorphology, however, often demonstrates significant inter‐observer variability, potentially resulting in missed or misdiagnosis. Developing an automated, accurate and universally applicable intelligent diagnosis system for APL would hold significant clinical importance by mitigating intra‐ and inter‐observer variability and enabling early diagnosis.

This study investigated the enhanced potential of artificial intelligence (AI)‐assisted morphology for the rapid screening and diagnosis of APL in daily batch specimens. The CELLSEE we proposed, an AI‐powered APL morphological diagnostic system featuring a convolutional neural network (CNN) with embedded attention mechanisms, which would recognise APL at 10× and 100× magnifications. Deep CNNs inherently integrate features across low, mid and high levels, and the ‘levels’ of features can be enriched by the number of stacked layers (depth).^7^ Conventional deep networks such as ResNet and VGGnet were utilised as the backbone network in CELLSEE system. Our model's architecture comprises three primary components: the backbone network, channel attention module and spatial attention module (Figure 1A). This study analysed 15 719 bone marrow smear (BMS) images from 83 confirmed APL cases and 118 randomly selected control samples, including acute and chronic leukaemia, roughly normal bone marrow and BMS samples after chemotherapy remission (Figure 1B). The diagnostic classification of leukaemia is based on 5th edition of World Health Organization classification of tumours of haematopoietic and lymphoid tissues, and AML risk stratification is carried out according to the 2022 European Leukemia Network guidelines. Detailed information on patient characteristics and cell image acquisition scheme has been provided (Table S1). The workflow was composed of three steps: pre‐processing, APL recognition and APL clinical diagnosis (Figure 1C). BMS images were analysed at 10× and/or 100× magnifications using Python, which employs a colour feature‐based segmentation method to delineate karyocyte regions in microscope image slices. Performance metrics for APL recognition were analysed and compared both at the image and patient levels (Table S2). Model evaluation was performed using area under the curve (AUC) and precision–recall (P–R) curves. A fivefold cross‐validation test was conducted to assess the model's robustness.

**FIGURE 1:**
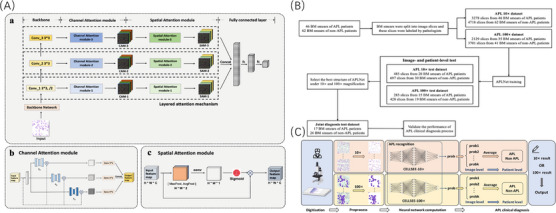
Architecture of the CELLSEE model, study design flowchart and APL clinical diagnosis process. (A) (a) CELLSEE comprises a backbone network, channel attention module, spatial attention module and two fully connected layers. The layered design facilitates the integration of abstract features extracted by deep neural networks and promotes feature fusion. (b) Architecture of the channel attention module. The multichannel attention–fusion (MCAF) module includes a channel attention weight module and feature fusion component. The channel attention weight module uses varying reduction ratios (r1, r2 and r3) for channel diversity, while the feature fusion component fuses three feature maps (head, body and tail) to generate the MCAF output. The MCAF aims to reassign channel weights with different reduction ratios and fuse features via multiple filter kernels. (c) Architecture of the spatial attention module. The module utilises max‐ and average‐pooling outputs pooled along the channel axis, which are then fed into a convolutional layer to obtain an output feature map. Applying pooling operations along the channel axis effectively highlights informative regions. (B) Study design flowchart. BMS from 83 confirmed APL and 118 control cases were used for the study. A dataset was established from 46 confirmed APL and 62 control cases BMS at 10× magnification, of which 35 confirmed APL and 41 control cases were used to create another dataset at 100× magnification. The performance of CELLSEE was evaluated using another 20 confirmed APL and 30 control cases BMS at 10× magnification, of which 15 confirmed APL and 19 control cases were analysed at 100× magnification. New BMS from 17 APL patients and 26 non‐APL patients were utilised to assess the performance of the joint diagnostic process. (C) The APL clinical diagnosis process flowchart encompasses digitisation, pre‐processing, neural network computation and diagnostic steps. The process can be divided into two main implementation paths: APL recognition at 10× and 100× magnification. Both pathways start by collecting BMS image slices in the monolayer region, which are fed into CELLSEE for patient‐level recognition and obtain the recognition results under 10× and 100× magnification images, respectively. APL, acute promyelocytic leukaemia; BMS, bone marrow smears.

The model was initially trained using the APL 10× dataset, and ResNet18, ResNet34 and ResNet50 were selected as the backbones of CELLSEE and denoted as CELLSEE18, CELLSEE34 and CELLSEE50. Subsequently, its performance was evaluated on image‐ and patient‐level, respectively (Tables S3 and S4). Loss values and accuracy metrics are plotted against epochs (Figure 2A,B). With an increase in epochs, both training and validation losses consistently decreased, while accuracy progressively increased. Approximately at epoch 50, the loss values for training and validation converged at .20 and .23, respectively, with accuracy reaching .98. The AUC for CELLSEE18 and CELLSEE50 exceeded that of their respective baseline networks, and the P–R curves further highlighted CELLSEE's superior performance, particularly CELLSEE50 (Figure 2C,D).

**FIGURE 2:**
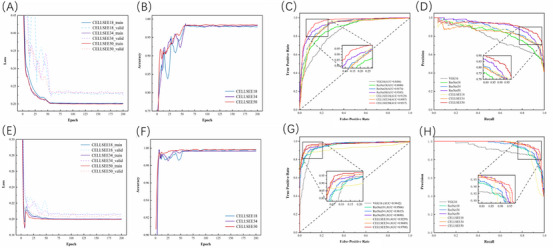
Loss and accuracy against epochs, and ROC and P–R curves for seven models trained on the APL 10× or 100× test datasets. (A, E) Training and validation losses against epochs on the APL 10× or 100× test datasets. As the number of epochs increased, the training and validation losses consistently decreased. (B, F) Accuracy against epochs on the APL 10× or 100× test datasets. With increasing epochs, the training and validation accuracy continue to increase. (C, G) ROC curve on the APL 10× or 100× test datasets. (D, I) P–R curve on the APL 10× or 100× test datasets. CELLSEE outperforms the baseline networks on both the APL 10× and 100× datasets. APL, acute promyelocytic leukaemia; P–R, precision–recall; ROC, receiver operating characteristic.

Furthermore, our model underwent training using the APL 100× dataset, followed by performance evaluation conducted at a 100× magnification on image‐level and exhibited greater robustness than at 10× magnification, with CELLSEE50 surpassing the other two CELLSEE models in terms of the three‐quarter metrics (Table S5). The loss value and accuracy are plotted against epochs (Figure 2E,F), showing similar trends to those observed in the results of 10× BMS images (Figure 2A,B). Upon closer comparison with the 10× results, the validation loss converged at .21 (an improvement from .23), and accuracy peaked at .99 (an enhancement from .98). Based on our fundamental understanding and supported by the performance evaluation of 100× BMS at the patient‐level (Table S6), it is clear that the application of high‐magnification BMS images significantly enhanced recognition performance. It is also evident that CELLSEE, trained with the APL 100× datasets, outperformed that trained using 10× images, exhibiting higher AUC values up to .9708 and more favourable P–R curve outcomes (Figure 2G,H). Furthermore, our model was compared with ResNet and VGG network and achieved superior image recognition performance. Among the seven models evaluated, CELLSEE50 emerged as the top performer, achieving an accuracy of up to 93.8% and a recall of 90.8% (Table S7).

We next focused on combining recognition under 10× and 100× magnifications to enhance the system's sensitivity in recognising APL while minimising the impact on accuracy. As expected in the performance evaluation of the joint diagnosis process, the combined approach at 10× and 100× magnifications yielded a recall value of 100.0%. In other words, as long as one of the pathways gives an APL judgement, the final recognition result will be viewed as APL patients. However, this was accompanied by a reduction in accuracy, precision and F1 score (Table S8). According to the definitions of metrics (Table S2), an increase in the number of false positives will lead to a decrease in accuracy and precision. Additionally, F1 score is an indicator that measures model P–R.

To investigate the effectiveness of the channel and spatial attention modules in the CELLSEE system, we selected CELLSEE50 as the model for conducting the ablation experiment on the APL 10× and 100× datasets due to its superior performance. The model CELLSEE50_NoCAM_10× represented the CELLSEE50 model without a channel attention module trained using 10× BMS images, while the model CELLSEE50_NoSAM_100× represented the CELLSEE50 model without a spatial attention module trained using 100× BMS images. The results demonstrated that the channel and spatial attention modules improved the accuracy, precision and F1 score for both 10× and 100× recognition (Table S9).

Neural networks are often referred to as ‘black boxes’ because the knowledge they learn is difficult to extract and present in a way that humans can understand. To address this issue, t‐distributed stochastic neighbour embedding (t‐SNE) and Gradient‐weighted Class Activation Mapping (Grad‐CAM) were employed in this study.^8^, ^9^ t‐SNE visualised CELLSEE's learned features to compare its learning ability when trained on 10× versus 100× images. The high‐dimensional features extracted by the last layer of CELLSEE were projected onto a two‐dimensional map (Figure 3A), elucidating why CELLSEE models exhibited superior classification ability when trained using the APL 100× dataset. Grad‐CAM is an algorithm for visualising deep neural network segments most contributory to predictions. The Grad‐CAM saliency map reveals that the karyocyte regions in the image were the key part of CELLSEE in recognising APL. This aligns with the visual cues used by doctors to identify APL, validating the correctness of the knowledge learned by the neural network (Figure 3B).

**FIGURE 3:**
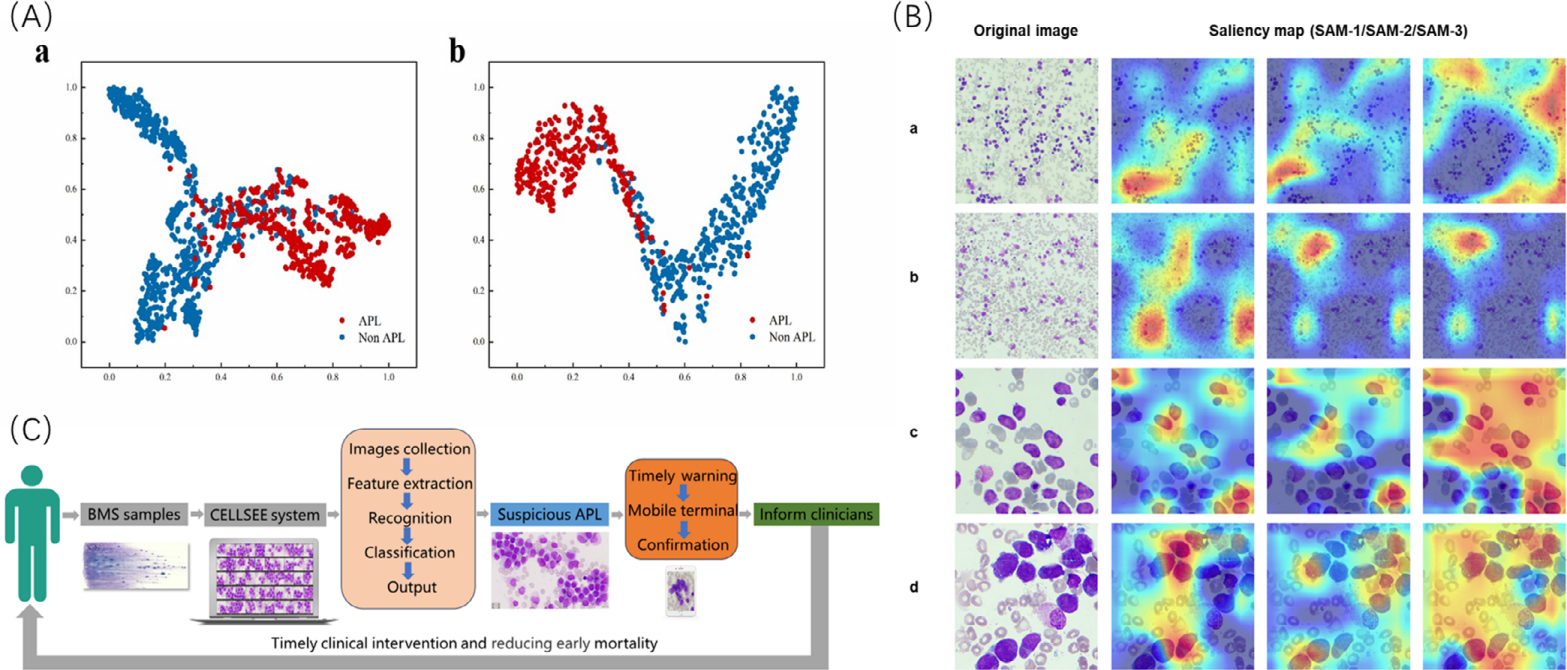
Visual algorithms of CELLSEE neural networks and overall process of APL warning and diagnosis. (A) CELLSEE's learned features were visualised with t‐SNE on APL test datasets to compare learning capabilities between models trained on 10× versus 100× images, thereby validating network performance. This indicated that the majority of promyelocytes from the APL patients have distinct features that differentiate them from other non‐APL patients. (a) 10× images, (b) 100× images. (B) CELLSEE's saliency maps, generated with the Grad‐CAM algorithm, illustrate pixel gradients in relation to CELLSEE's loss function. Brighter pixels indicate a greater influence on CELLSEE's classification. (a, b) 10× images, (c, d) 100× images. (C) The APL warning and diagnostic process. The CELLSEE system batch detects BMS samples, instantly flags suspected APL cases, and forwards them to a haematologist's mobile terminal for confirmation. Results are promptly reported to clinicians, enabling timely treatment for APL patients and contributing to a reduction in early mortality. APL, acute promyelocytic leukaemia; Grad‐CAM, Gradient‐weighted Class Activation Mapping ; t‐SNE, t‐distributed stochastic neighbour embedding.

Overall, our study presents an AI‐assisted end‐to‐end workflow for the rapid detection of APL in batch specimens. The workflow starts with the prompt collection of stained bone marrow images at 10× and 100× magnifications, followed by instant image screening to detect suspected APL. Suspected images are then sent to a designated pathologist's mobile terminal for final diagnosis, regardless of their location. Compared with traditional microscopy methods, the CELLSEE system enables immediate judgement following image acquisition, notably during nonworking hours, and circumvents delays in APL diagnosis caused by manual testing queues (Figure 3C). Due to APL's specific genetic abnormality t(15;17), our work also indicates the potential of using AI morphology to more precisely predict leukaemia with defining genetic abnormalities, thereby narrowing the scope of genetic abnormalities screening in newly diagnosed leukaemia patients. These aspects will be further explored in our on‐going research on AI morphology diagnosis in patients with AML harbouring the *RUNX1::RUNX1T1* fusion gene.

## AUTHOR CONTRIBUTIONS

Gusheng Tang, Chun Xu and Yang Song designed the study. Jun Wu, Yan Zhang, Yuanyuan Yang and Fangyu Guo collected bone marrow smear samples. Yujun Xiao and Zheng Huang analysed the data. Hui Cheng wrote the manuscript. Xiong Ni, Xinhua Hu and Jianmin Yang reviewed manuscript. All authors approved the final version of the manuscript before submission.

## CONFLICT OF INTEREST STATEMENT

The authors declare no conflicts of interest.

## ETHICS STATEMENT

The study was conducted in accordance with the Declaration of Helsinki and approved by the Ethics Committee of Shanghai Changhai Hospital.

## Supporting information

Supporting Information

Supporting Information

Supporting Information

Supporting Information

Supporting Information

Supporting Information

Supporting Information

Supporting Information

Supporting Information

## Data Availability

All images and raw data for statistical analysis in this study are available from the corresponding author upon reasonable request.
