# Supplementary material for: Rapid screening of acute promyelocytic leukaemia in daily batch specimens: A novel artificial intelligence‐enabled approach to bone marrow morphology
Source: Clin Transl Med. 2024 Jul 23;14(7):e1783. doi: 10.1002/ctm2.1783 (PMC11263731; doi:10.1002/ctm2.1783)
Supplement: Supplementary file 1 — Supporting Information [file CTM2-14-e1783-s007.docx]

| Parameter | APL | non-APL AML | ALL | CML | CLL | Normal bone marrow | CR |
| --- | --- | --- | --- | --- | --- | --- | --- |
| n | 83 | 62 | 18 | 10 | 8 | 10 | 10 |
| Age, median | 46 (30-72) | 54.5(21-79) | 32(14-56) | 37.5(23-71) | 42(38-70) | 29(18-62) | 34(22-64) |
| *Sex, %* |  |  |  |  |  |  |  |
| Male | 47.7 | 51.4 | 60.2 | 58.9 | 52.5 | 46.5 | 45.5 |
| Female | 52.3 | 48.6 | 39.8 | 41.1 | 47.5 | 53.5 | 54.5 |
| WBC (×10^9^), median | 12.41(1.82-79.43) | 13.56(3.72-149.62) | 8.37(4.36-82.30) | 41.56(18.25-237.64) | 15.50(12.43-69.02) | 6.58(3.25-13.09) | 5.37(2.34-8.71) |
| *Karyotype, %* |  |  |  |  |  |  |  |
| t (15;17) | 100 | / | / | / | / | / | / |
| t (8;21) | / | 10.4 | / | / | / | / | / |
| inv (16) or t (16;16) | / | 9.8 | / | / | / | / | / |
| t (9;22) | / | / | 21.5 | 100 | / | / | / |
| Other complex karyotype | / | 13.7 | 10.5 | / | 24.5 | / | / |
| Normal karyotype | / | 66.1 | 68 | / | 75.5 | / | / |
| *Fusion gene, %* |  |  |  |  |  |  |  |
| PML-RARα | 100 | / | / | / | / |  |  |
| RUNX1-RUNX1T1 | / | 10.4 | / | / | / | / | / |
| CBFβ-MYH11 | / | 9.8 | / | / | / | / | / |
| BCR-ABL | / | / | 21.5 | 100 | / | / | / |
| MLL arrangement | / | 5.2 | 4.7 | / | / | / | / |
| No fusion gene | / | 74.6 | 73.8 | / | 100 | / | / |
| *2022 ELN Risk stratification* |  |  |  |  |  |  |  |
| Favorable | / | 24.3 | / | / | / | / | / |
| Intermediate | / | 52.5 | / | / | / | / | / |
| Adverse | / | 23.2 | / | / | / | / | / |
| *Training dataset, n* |  |  |  |  |  |  |  |
| 10× | 46 | 37 | 8 | 5 | 4 | 4 | 4 |
| 100× | 35 | 25 | 5 | 3 | 2 | 2 | 4 |
| *Test dataset, n* |  |  |  |  |  |  |  |
| 10× | 20 | 15 | 6 | 3 | 2 | 2 | 2 |
| 100× | 15 | 10 | 3 | 2 | 1 | 2 | 1 |
| *Joint diagnosis, n* |  |  |  |  |  |  |  |
| 10×and 100× | 17 | 10 | 4 | 2 | 2 | 4 | 4 |

**Table S1**. Patient characteristics and cell image acquisition scheme

Patient characteristics of APL and control (non-APL AML, ALL, CML, CLL, relative normal bone marrow and CR) groups. AML type was defined according to the WHO 2022 classification. Bone marrow smear (BMS) specimens from a total of 83 confirmed APL and 118 control cases were used for the study. Training dataset was established from 46 confirmed APL and 62 control cases BMS at 10× magnification, of which 35 confirmed APL and 41 control cases were used to create another dataset at 100× magnification. The performance of CELLSEE was evaluated using another 20 confirmed APL and 30 control cases BMS at 10× magnification, of which 15 confirmed APL and 19 control cases were analyzed at 100× magnification. New BMS from 17 APL patients and 26 non-APL patients were utilized to assess the performance of the joint diagnostic process. APL Acute promyelocytic leukemia, AML Acute myeloid leukemia, ALL Acute lymphoblastic leukemia, CML Chronic myeloid leukemia, CLL Chronic lymphocyte leukemia, CR Complete remission after treatment, WBC White blood cell count.
