# Supplementary material for: Rapid screening of acute promyelocytic leukaemia in daily batch specimens: A novel artificial intelligence‐enabled approach to bone marrow morphology
Source: Clin Transl Med. 2024 Jul 23;14(7):e1783. doi: 10.1002/ctm2.1783 (PMC11263731; doi:10.1002/ctm2.1783)
Supplement: Supplementary file 2 — Supporting Information [file CTM2-14-e1783-s002.docx]

**Table S2.** The definitions of the metrics.

| Metric | Definition |
| --- | --- |
| Accuracy | $Accuracy=\frac{TP+TN}{TP+FP+TN+FN}$ |
| True Positive Rate / Recall | $TPR/Recall=\frac{TP}{TP+FN}$ |
| False Positive Rate | $FPR=\frac{FP}{TN+FP}$ |
| Precision | $Precision=\frac{TP}{TP+FP}$ |
| F1 | $F1=2\frac{Precision\times Recall}{Precision+Recall}$ |
