# Supplementary material for: Rapid screening of acute promyelocytic leukaemia in daily batch specimens: A novel artificial intelligence‐enabled approach to bone marrow morphology
Source: Clin Transl Med. 2024 Jul 23;14(7):e1783. doi: 10.1002/ctm2.1783 (PMC11263731; doi:10.1002/ctm2.1783)
Supplement: Supplementary file 3 — Supporting Information [file CTM2-14-e1783-s009.docx]

**Table S3.** The image-level performance of three CELLSEE models on the APL 10× dataset by 5-fold cross-validation.

| Model | Accuracy | Precision | Recall | F1 | NPV |
| --- | --- | --- | --- | --- | --- |
| CELLSEE18 | 0.8390±0.0069 | 0.7857±0.0160 | 0.8360±0.0195 | 0.8100±0.0079 | 0.8809±0.0106 |
| CELLSEE34 | 0.8291±0.0107 | 0.7649±0.0160 | 0.8453±0.0170 | 0.7912±0.0128 | 0.8630±0.0215 |
| CELLSEE50 | 0.8933±0.0122 | 0.8445±0.0235 | 0.9079±0.0115 | 0.8749±0.0132 | 0.9322±0.0082 |
