# Supplementary material for: Rapid screening of acute promyelocytic leukaemia in daily batch specimens: A novel artificial intelligence‐enabled approach to bone marrow morphology
Source: Clin Transl Med. 2024 Jul 23;14(7):e1783. doi: 10.1002/ctm2.1783 (PMC11263731; doi:10.1002/ctm2.1783)
Supplement: Supplementary file 4 — Supporting Information [file CTM2-14-e1783-s008.docx]

**Table 4.** The patient-level performance of three CELLSEE models on the APL 10× dataset.

| Model | Accuracy | Precision | Recall | F1 | NPV |
| --- | --- | --- | --- | --- | --- |
| CELLSEE18 | 0.8449±0.0344 | 0.7543±0.0433 | 0.9111±0.0415 | 0.8249±0.0371 | 0.9358±0.0269 |
| CELLSEE34 | 0.8450±0.0056 | 0.8075±0.0087 | 0.8173±0.0199 | 0.8122±0.0086 | 0.9191±0.0345 |
| CELLSEE50 | 0.9029±0.0229 | 0.8389±0.0408 | 0.9397±0.0250 | 0.8862±0.0249 | 0.9596±0.0115 |
