# Supplementary material for: Rapid screening of acute promyelocytic leukaemia in daily batch specimens: A novel artificial intelligence‐enabled approach to bone marrow morphology
Source: Clin Transl Med. 2024 Jul 23;14(7):e1783. doi: 10.1002/ctm2.1783 (PMC11263731; doi:10.1002/ctm2.1783)
Supplement: Supplementary file 5 — Supporting Information [file CTM2-14-e1783-s005.docx]

**Table S5.** The image-level performance of three CELLSEE models on the APL 100× dataset by 5-fold cross-validation.

| Model | Accuracy | Precision | Recall | F1 | NPV |
| --- | --- | --- | --- | --- | --- |
| CELLSEE18 | 0.9126±0.0026 | 0.9189±0.0151 | 0.8555±0.0178 | 0.8860±0.0041 | 0.9090±0.0092 |
| CELLSEE34 | 0.9354±0.0042 | 0.9435±0.0134 | 0.8916±0.0191 | 0.9168±0.0061 | 0.9214±0.0109 |
| CELLSEE50 | 0.9400±0.0044 | 0.9408±0.0029 | 0.9058±0.0175 | 0.9228±0.0062 | 0.9395±0.0118 |
