# Supplementary material for: Rapid screening of acute promyelocytic leukaemia in daily batch specimens: A novel artificial intelligence‐enabled approach to bone marrow morphology
Source: Clin Transl Med. 2024 Jul 23;14(7):e1783. doi: 10.1002/ctm2.1783 (PMC11263731; doi:10.1002/ctm2.1783)
Supplement: Supplementary file 6 — Supporting Information [file CTM2-14-e1783-s001.docx]

**Table S6.** The patient-level performance of three CELLSEE models on the APL 100× dataset.

| Model | Accuracy | Precision | Recall | F1 | NPV |
| --- | --- | --- | --- | --- | --- |
| CELLSEE18 | 0.9305±0.0223 | 0.9493±0.0320 | 0.8900±0.0376 | 0.9186±0.0266 | 0.9205±0.0234 |
| CELLSEE34 | 0.9418±0.0345 | 0.8949±0.0546 | 0.9862±0.0274 | 0.9381±0.0357 | 0.9913±0.0200 |
| CELLSEE50 | 0.9516±0.0255 | 0.9282±0.0205 | 0.9644±0.0392 | 0.9458±0.0255 | 0.9718±0.0273 |
