# Supplementary material for: Rapid screening of acute promyelocytic leukaemia in daily batch specimens: A novel artificial intelligence‐enabled approach to bone marrow morphology
Source: Clin Transl Med. 2024 Jul 23;14(7):e1783. doi: 10.1002/ctm2.1783 (PMC11263731; doi:10.1002/ctm2.1783)
Supplement: Supplementary file 7 — Supporting Information [file CTM2-14-e1783-s003.docx]

**Table S7.** The image-level performance of the seven models on the APL 100× dataset.

| Model | Accuracy | Precision | Recall | F1 | NPV |
| --- | --- | --- | --- | --- | --- |
| VGG16 | 0.886 | 0.853 | 0.862 | 0.858 | 0.908 |
| ResNet18 | 0.895 | 0.923 | 0.802 | 0.858 | 0.880 |
| ResNet34 | 0.909 | 0.884 | 0.887 | 0.885 | 0.925 |
| ResNet50 | 0.928 | 0.923 | 0.894 | 0.908 | 0.931 |
| CELLSEE18 | 0.921 | 0.960 | 0.837 | 0.894 | 0.901 |
| CELLSEE34 | 0.932 | 0.927 | 0.901 | 0.914 | 0.936 |
| CELLSEE50 | 0.938 | 0.935 | 0.908 | 0.921 | 0.940 |
