# Supplementary material for: Rapid screening of acute promyelocytic leukaemia in daily batch specimens: A novel artificial intelligence‐enabled approach to bone marrow morphology
Source: Clin Transl Med. 2024 Jul 23;14(7):e1783. doi: 10.1002/ctm2.1783 (PMC11263731; doi:10.1002/ctm2.1783)
Supplement: Supplementary file 8 — Supporting Information [file CTM2-14-e1783-s006.docx]

**Table S8.** The performance of CELLSEE50 in the joint APL clinical diagnosis process, including the detailed performance of the 10× and 100× implementation paths.

| Method | Accuracy | Precision | Recall | F1 | NPV |
| --- | --- | --- | --- | --- | --- |
| 10× implementation path | 0.930 | 0.889 | 0.941 | 0.914 | 0.960 |
| 100× implementation path | 0.953 | 0.941 | 0.941 | 0.941 | 0.962 |
| Joint diagnosis process | 0.930 | 0.850 | 1.000 | 0.919 | 1.000 |
