# Supplementary material for: Rapid screening of acute promyelocytic leukaemia in daily batch specimens: A novel artificial intelligence‐enabled approach to bone marrow morphology
Source: Clin Transl Med. 2024 Jul 23;14(7):e1783. doi: 10.1002/ctm2.1783 (PMC11263731; doi:10.1002/ctm2.1783)
Supplement: Supplementary file 9 — Supporting Information [file CTM2-14-e1783-s004.docx]

**Table S9.** The performance of ablation experiment on APL 10× and 100× dataset by 5-fold cross-validation.

| Model | Accuracy | Precision | Recall | F1 | NPV |
| --- | --- | --- | --- | --- | --- |
| CELLSEE50_10× | 0.8933±0.0122 | 0.8445±0.0235 | 0.9079±0.0115 | 0.8749±0.0132 | 0.9322±0.0082 |
| CELLSEE50_NoCAM_10× | 0.8790±0.0044 | 0.8180±0.0100 | 0.9080±0.0108 | 0.8606±0.0040 | 0.9310±0.0082 |
| CELLSEE50_NoSAM_10× | 0.8789±0.0104 | 0.8159±0.0151 | 0.9106±0.0087 | 0.8606±0.0107 | 0.9303±0.0071 |
| CELLSEE50_100× | 0.9400±0.0044 | 0.9408±0.0090 | 0.9058±0.0175 | 0.9228±0.0062 | 0.9395±0.0118 |
| CELLSEE50_NoCAM_100× | 0.9333±0.0061 | 0.9245±0.0229 | 0.9069±0.0141 | 0.9157±0.0065 | 0.9334±0.0181 |
| CELLSEE50_NoSAM_100× | 0.9281±0.0141 | 0.9200±0.0268 | 0.8978±0.0322 | 0.9086±0.0177 | 0.9393±0.0078 |
